# Supplementary material for: Correlation and shear bands in a plastically deformed granular medium
Source: Sci Rep. 2018 Mar 5;8:4021. doi: 10.1038/s41598-018-22310-z (PMC5838169; doi:10.1038/s41598-018-22310-z)
Supplement: Supplementary file 1 — Supplementary Information [file 41598_2018_22310_MOESM1_ESM.pdf]

Supplementary Materials: Correlation and shear bands in a plastically deformed granular medium

Kamran Karimi
Université Grenoble Alpes, CNRS, ISTerre, 38041 Grenoble cedex 9, France

Jean-Louis Barrat
Université Grenoble Alpes, CNRS, LIPHY, F-38000 Grenoble, France

I. SHEAR BAND VISUALISATION

The activity map featured in the movie (click here) evolves toward multiple localization bands as the loading continues (the imposed strain is also indicated). The color map is based on the height of each active site. The data was visualized by OVITO [1].

II. DERIVATION OF THE OSEEN TENSOR FOR A COMPRESSIBLE MEDIUM

The force balance equation in a continuum reads

$$\partial_\alpha \sigma_{\alpha\beta} + f_\beta = 0, \tag{1}$$

where $\sigma_{\alpha\beta}$ is the stress tensor and f_β is the applied force per unit volume. Rewriting the above equation in the q-space, it follows that

$$iq_\alpha \; \bar{\sigma}_{\alpha\beta} + \bar{f}_\beta = 0, \tag{2}$$

where q_α is the (longitudinal) wave vector. Here the bars denote the transformed fields.

In a linear isotropic homogeneous elastic medium

$$\bar{\sigma}_{\alpha\beta} = \lambda \bar{\epsilon}_{\gamma\gamma}\delta_{\alpha\beta} + 2\mu\bar{\epsilon}_{\alpha\beta}, \tag{3}$$

with λ and μ being the Lamé constants and the strain tensor $\bar{\epsilon}_{\alpha\beta}$ is

$$\bar{\epsilon}_{\alpha\beta} = \frac{i}{2}(q_\alpha \; \bar{u}_\beta + q_\beta \; \bar{u}_\alpha), \tag{4}$$

where $\bar{u}_\alpha$ denotes the displacement vector. Inserting Eq. 3 into Eq. 2 and using Eq. 4, it follows that

$$\begin{aligned} O^{-1}_{\alpha\beta}\bar{u}_\beta &= \bar{f}_\beta, \\ O^{-1}_{\alpha\beta} &= (\lambda+\mu)q_\alpha q_\beta + \mu q^2 \delta_{\alpha\beta}, \end{aligned} \tag{5}$$

where the Oseen tensor $O_{\alpha\beta}$ represents a general Green's function of the problem and $q^2 = q_\alpha q_\alpha$. The above tensorial form can be recast in two dimensions as the following

$$O^{-1}_{\alpha\beta} = (\lambda+2\mu)q_\alpha q_\beta + \mu q^\perp_\alpha q^\perp_\beta, \tag{6}$$

or

$$q^4 O_{\alpha\beta} = (\lambda+2\mu)^{-1} q_\alpha q_\beta + \mu^{-1} q^\perp_\alpha q^\perp_\beta, \tag{7}$$

where $q^\perp_\alpha$ is the transverse wave vector. The inversion was performed on the grounds that longitudinal and transverse waves emerge as the eigenmodes of the linear operator.

Let the bulk modulus be $K = \lambda + \mu$ in two dimensions. Therefore,

$$O_{\alpha\beta} = \frac{1}{\mu q^2}(\delta_{\alpha\beta} - \frac{\hat{q}_\alpha \hat{q}_\beta}{1+\frac{\mu}{K}}), \tag{8}$$

with $\hat{q}_\alpha = \frac{q_\alpha}{q}$ and $\delta_{\alpha\beta}$ being the Kronecker delta. An effective source due to a localized distortion is expressed as

$$\begin{aligned} f_\beta &= -\partial_\alpha(2\mu\epsilon^{\mathrm{stz}}_{\alpha\beta}), \\ \epsilon^{\mathrm{tz}}_{\alpha\beta} &= \epsilon^*(\delta_{x\alpha}\delta_{x\beta} - \delta_{y\alpha}\delta_{y\beta})a^d\delta(r), \end{aligned} \tag{9}$$

where ϵ^* is the total released strain over the local volume a^d and $\delta(...)$ denotes the delta function. Therefore,

$$\begin{aligned} \bar{f}_\beta &= -iq_\alpha(2\mu\bar{\epsilon}^{\mathrm{stz}}_{\alpha\beta}) \\ &= -i(2\mu\epsilon^*)a^d(q_x\delta_{x\beta} - q_y\delta_{y\beta}). \end{aligned} \tag{10}$$

Using Eqs. 8 and 10 gives

$$\begin{aligned} \bar{u}_\alpha &= O_{\alpha\beta}\bar{f}_\beta \\ &= -2i\epsilon^* a^d q^{-2}[(q_x\delta_{\alpha x} - q_y\delta_{\alpha y}) \\ &\quad - (1+\frac{\mu}{K})^{-1}q^{-2}(q_x^2 - q_y^2)q_\alpha]. \end{aligned} \tag{11}$$

Making use of Eqs. 3 and 4 allows for the stress purturbation fields to be determined as follows

$$\begin{aligned} \bar{p} &= -K(iq_\alpha\bar{u}_\alpha) \\ &= -\frac{2\mu\epsilon^*}{1+\frac{\mu}{K}}a^d\cos 2\theta, \\ \bar{\sigma} &= i\mu(q_x\bar{u}_x - q_y\bar{u}_y) \\ &= 2\mu\epsilon^* a^d - \frac{2\mu\epsilon^*}{1+\frac{\mu}{K}}a^d\frac{1}{2}(1+\cos 4\theta), \end{aligned} \tag{12}$$

where θ is the angle of the wave vector q_α.

For a localized dilation $\epsilon^{\mathrm{tz}}_{\alpha\beta} = \frac{1}{2}\epsilon^*_v\delta_{\alpha\beta}a^d\delta(r)$ with the released volumetric strain ϵ^*_v , we obtain

$$\begin{aligned} \bar{p} &= -\frac{K\epsilon^*_v}{1+\frac{\mu}{K}}a^d, \\ \bar{\sigma} &= \frac{\mu\epsilon^*_v}{1+\frac{\mu}{K}}a^d\cos 2\theta. \end{aligned} \tag{13}$$

[1] A. Stukowski, Modelling and Simulation in Materials Science and Engineering **18**, 015012 (2009).
